# Neural effects of multisensory dance training in Parkinson’s disease: evidence from a longitudinal neuroimaging single case study

**DOI:** 10.3389/fnagi.2024.1398871

**Published:** 2024-10-09

**Authors:** Jenny R. Simon, Judith Bek, Katayoun Ghanai, Karolina A. Bearss, Rebecca E. Barnstaple, Rachel J. Bar, Joseph F. X. DeSouza

**Affiliations:** ^1^Department of Psychology, Centre for Vision Research, York University, Toronto, ON, Canada; ^2^Faculty of Kinesiology and Physical Education, Centre for Motor Control, University of Toronto, Toronto, ON, Canada; ^3^School of Psychology, University College Dublin, Dublin, Ireland; ^4^Department of Music, Neuroscience Graduate Diploma Program, York University, Toronto, ON, Canada; ^5^School of English and Theatre Studies, College of Arts University of Guelph, Guelph, ON, Canada; ^6^Canada’s National Ballet School, Toronto, ON, Canada; ^7^Multisensory Neuroscience Laboratory, VISTA, Connected Minds, Canadian Action and Perception Network (CAPnet), York University, Toronto, ON, Canada

**Keywords:** Parkinson’s disease, fMRI, dance, neurorehabilitation, neuroplasticity, learning, motor imagery

## Abstract

Dance is associated with beneficial outcomes in motor and non-motor domains in Parkinson’s disease (PD) and regular participation may help delay symptom progression in mild PD. However, little is known about the neurobiological mechanisms of dance interventions for PD. The present case study explored potential neuroplastic changes in a 69-year-old male with mild PD participating in regular dance classes over 29 weeks. Functional MRI was performed at four timepoints (pre-training, 11 weeks, 18 weeks, 29 weeks), where the individual imagined a dance choreography while listening to the corresponding music. Neural activity was compared between dance-imagery and fixation blocks at each timepoint. Analysis of functionally defined regions revealed significant blood-oxygen-level-dependent (BOLD) signal activation in the supplementary motor area, right and left superior temporal gyri and left and right insula, with modulation of these regions observed over the training period except for the left insula. The results suggest the potential for dance to induce neuroplastic changes in people with PD in regions associated with motor planning and learning, auditory processing, rhythm, emotion, and multisensory integration. The findings are consistent with dance being a multimodal therapeutic activity that could provide long-term benefits for people with PD.

## 1 Introduction

Parkinson’s disease (PD) is a neurodegenerative condition in which a loss of dopamine-producing neurons in the substantia nigra pars compacta leads to motor symptoms such as bradykinesia, tremor, rigidity, and postural instability. People living with PD also experience a range of non-motor symptoms including depression, anxiety, and apathy (Poewe, 2008). Currently PD is incurable, but treatments such as dopaminergic medication and deep brain stimulation (DBS) can help to alleviate symptoms and improve quality of life (Lee and Yankee, 2022). However, while medical and surgical treatments can be effective, they are associated with risks and side effects. For example, levodopa, the most common medication for PD, improves motor impairments but can lead to structural alterations in the brain causing complications such as dyskinesia (Ogawa et al., 2021). Additionally, while DBS can be effective for some individuals, costs of surgery are substantial and intensive post-surgery care is needed (Lozano et al., 2019; Erdem et al., 2022). The importance of non-invasive and non-pharmacologic approaches such as physiotherapy, exercise, and dance is increasingly recognized, particularly as the burden to healthcare systems continues to grow at an unsustainable rate with the increasing prevalence of PD (Dorsey et al., 2018).

Dance is a complex form of human movement which activates an intricate network of regions in the brain (Dhami et al., 2015). Engaging these areas of the brain through dance training facilitates structural and functional changes assumed to reflect more efficient functioning in expert dancers (Bar and DeSouza, 2016; Burzynska et al., 2017). Numerous studies have associated dance with improvements in motor functioning as well as non-motor symptoms in people with PD (for reviews see Bek et al., 2020; Kshtriya et al., 2015). Although few studies have examined the effects of long-term dance training in PD, a recent 3-year longitudinal study found evidence that regular dance participation may delay progression of motor and non-motor symptoms in people with mild PD (Bearss and DeSouza, 2021).

Dance training has been found to promote neural plasticity in professional dancers (Bar and DeSouza, 2016; Burzynska et al., 2017). For example, Bar and DeSouza (2016) found that learning a new dance choreography over 8 months was associated with a significant decrease in activation in the supplementary motor area (SMA) and the left and right auditory cortices. In older adults, behavioral changes resulting from dance training have been associated with structural and functional changes in the brain such as increased functional connectivity, increased white matter integrity, and increased volume in cognitive and motor regions (Rehfeld et al., 2018; Teixeira-Machado et al., 2019; Balazova et al., 2021; Meulenberg et al., 2023).

A recent study investigating the neuroplastic effects of dance for people with PD found changes in activation in areas of the motor cortex and cerebellum after 12 weeks of a Tango intervention (Kashyap et al., 2021). A previous single case study of an individual with PD also found an increase in functional connectivity between the basal ganglia and premotor cortex after 5 days of dance training (Batson et al., 2014). However, the neural mechanisms underlying motor improvement through long-term dance participation remain largely under-investigated in people living with PD (Meulenberg et al., 2023).

The present case study reports a longitudinal investigation of an individual with PD participating in regular dance classes over a period of 29 weeks, as part of a larger study to identify potential indicators of neuroplastic changes associated with dance training (Bearss and DeSouza, 2021). Functional magnetic resonance imaging (fMRI) was performed at four timepoints across the training period, to examine modulation of cortical activity when the individual imagined dance while listening to the music associated with the learned choreography.

## 2 Methods

### 2.1 Participant characteristics

The participant was a 69-year-old male with mild idiopathic PD with disease duration of 4 years and a Unified Parkinson’s Disease Rating Scale (MDS-UPDRS) (Goetz et al., 2007) motor score of 12, who was taking dopaminergic medication at the time of the study. The participant was left-handed according to the Edinburgh Handedness Inventory (Oldfield, 1971). In addition to participation in weekly dance classes, the participant also reported regularly walking 6 miles per week. The participant had no previous dance experience. The study was approved by the Office of Research Ethics committee at York University (REB#2013–211). All procedures were conducted in accordance with the requirements of the ethical approval and the Declaration of Helsinki. The participant provided written informed consent prior to the data collection.

### 2.2 Dance training

The participant attended weekly specialist dance classes (75 min in duration) taught by Dance for PD trained instructors at Canada’s National Ballet School (NBS) in Toronto, Ontario. Classes were attended by an average of 20 people with PD, with 15–20 trained volunteers assisting as needed. The dance classes included elements of jazz steps, ballet, Argentinian tango, dance theater, freestyle and choreographed movements, accompanied by live music from a pianist. Each class included a warm-up followed by seated and standing exercises, before practicing a 2-min choreography facing a partner (see Supplementary Table S1, for an example dance class outline). Sections of the choreography were based on a narrative, which the instructor would first describe before demonstrating the movements. A video illustrating part of the choreography is available at: https://bit.ly/42cMlth. While no structured training was provided outside of classes, the music accompanying the choreography was provided and the participant reported self-directed practice of the dance at home for approximately 4.5 h per week as well as 0.5–1 h per week imagining the dance with and without physical practice.

### 2.3 Scanning procedure

The participant underwent a series of 4 fMRI scanning sessions over a period of 29 weeks. Prior to each scanning session, safety screening was completed and training for the dance-imagery task was provided.

A 3 T Siemens Tim Trio MRI scanner was used to acquire functional and anatomical images using a 32-channel head coil. T2*-weighted echo planar imaging was performed using parallel imaging (GRAPPA) with an acceleration factor of 2X with the following parameters: 32-slices, 56 × 70 matrix, 210 mm × 168 mm FOV, 3 × 3 × 4 mm slice thick, TE = 30 ms, flip angle of 90°, volume acquisition time of 2,000 ms. Each scan consisted of 240 volumes. Echo-planar images were co-registered with the high-resolution (1 mm^3^) anatomical scan of the participant’s brain taken at the end of each session (spin echo, TR = 1,900 ms, TE = 2.52 ms, flip angle = 9°, 256 × 256 matrix). The participant’s head was secured in place with cushions to minimize movements.

While in the scanner, the participant was instructed to imagine the choreography practiced during training in the dance studio, from a first-person perspective (including both visual and kinesthetic modalities), while the first minute of the music associated with the 2-min choreography was played through headphones. A block-design was employed where 60 s of the dance-imagery task (ON state) alternated with fixation blocks of 30 s (OFF state). These blocks were alternated and repeated five times for both blocks with a total scan time of 8 min (the first 15 volumes are not included in the analysis). The four timepoints were: pre-training (T1), where the participant had only attended one class with the music and choreography to be learned; after 11 weeks of training (T2); after 18 weeks of training (T3) and after 29 weeks of training (T4).

### 2.4 Data processing and analysis

Processing and analysis of the fMRI data was conducted using BrainVoyager QX (version 22.4.2, Brain Innovation, Maastricht, The Netherlands). Functional data were superimposed on an anatomical scan and transformed into Talairach space. Pre-processing steps (slice time correction, motion correction, and temporal high pass filtering) were applied to all runs. To account for periodic fluctuations in the fMRI signal, a General Linear Model (GLM) incorporating Fourier basis functions was applied, including two sine and two cosine functions to model low-frequency drifts and physiological noise. Across the four timepoints, the maximum motion correction did not exceed 1 mm for translation and 3 mm for rotation. None of the scans was excluded because of head movements. A fixed effects single-subject (FFX GLM) analysis was subsequently performed to compare activity during the dance-imagery blocks and the fixation blocks within each timepoint (T1, T2, T3, and T4). Functionally defined regions were identified from the GLM of dance-imagery versus fixation across all four timepoints, according to a statistical threshold of *p* < 0.0001 (Bonferroni-corrected) with a cluster threshold of k > 22. The BOLD percent signal change was calculated relative to a baseline, defined as the average of the two volumes acquired prior to the start of the music.

Modulation of task-related BOLD signal change (dance-imagery vs. fixation) was compared between timepoints for each of the functionally defined regions, using linear mixed-effects models to examine the effect of Time on percentage BOLD signal change associated with the dance-imagery task. The statistical models included Time (T1/T2/T3/T4) as a fixed factor with T1 as the reference level, as well as random effects for individual samples. An autoregressive correlation structure of order 1 [AR (1)] was included to account for correlations between consecutive samples. An adjusted significance threshold of *p* < 0.0033 was used to correct for multiple comparisons within and between models. Statistical analyses were conducted in R (R Core Team, 2023).

## 3 Results

**Figure 1:**
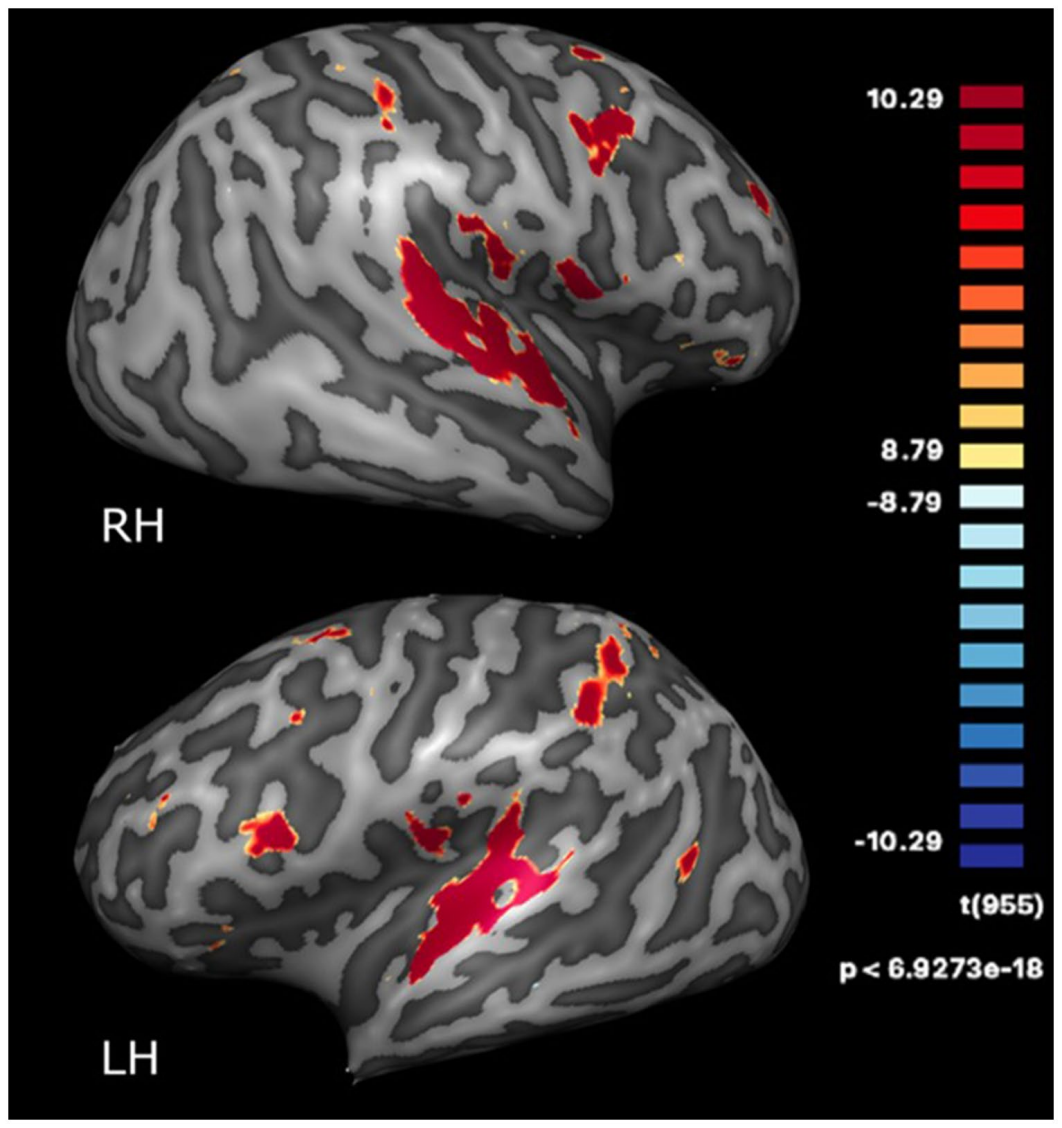
Regions activated during the dance-imagery task (vs. fixation) projected onto the inflated cortex of the right (RH) and left (LH) hemispheres, displayed at a statistical threshold of *p* < 0.0001, Bonferroni-corrected, cluster threshold k > 22.

Functionally defined regions based on clusters of functional activation across all four timepoints were identified from the GLM. These regions were SMA, left and right superior temporal gyrus (STG), and left and right insula, which were significantly activated at a statistical threshold of *p* < 0.0001 (Bonferroni-corrected) with a cluster threshold of k > 22 (see Figure 1). Talairach coordinates for these regions are provided in Supplementary Table S2.

**Figure 2:**
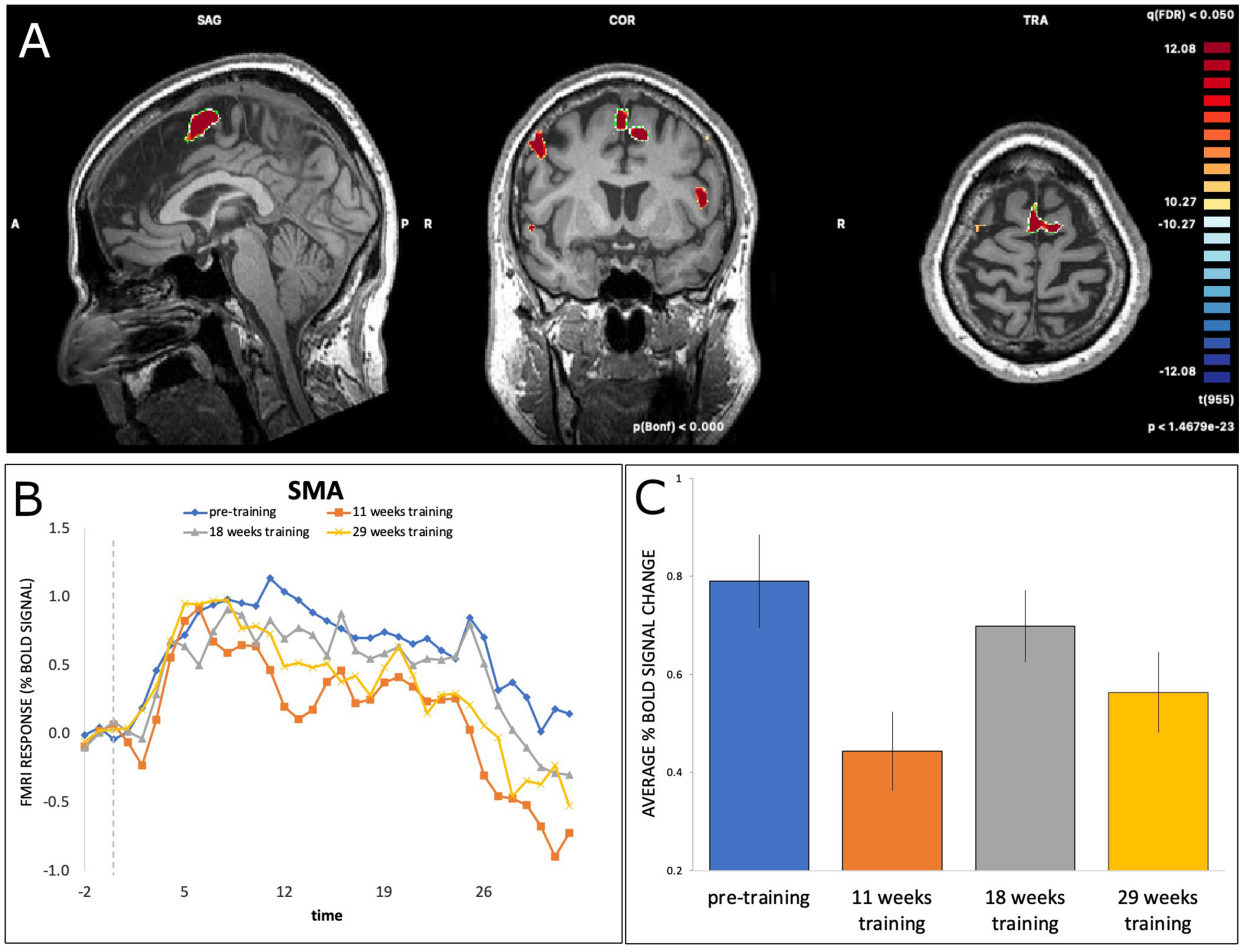
**(A)** BOLD activation of SMA during dance-imagery shown in sagittal, coronal, and transverse view in the 3D Talairach space, displayed at a statistical threshold of *p* < 0.0001, Bonferroni-corrected, with cluster threshold k > 22; **(B)** fMRI response (average percent BOLD signal change) of SMA during the dance-imagery blocks within each of the four timepoints (dashed line indicates the start of the music played in the scanner); **(C)** average percent BOLD signal change of SMA between the four timepoints. Error bars represent S.E.M.

Linear mixed-effect modelling revealed significant modulation of BOLD signal activation in the SMA (using the adjusted significance threshold) at T2 (*b* = −0.35, SE = 0.055, *t*(413) = −6.32, *p* < 0.001) and at T4 (*b* = −23, SE = 0.054, *t*(413) = −4.19, *p* < 0.001), but not at T3 (*b* = −0.09, SE = 0.054, *t*(413) = −1.70, *p* = 0.09), relative to T1. As illustrated in Figure 2, activation decreased from T1 (pre-training) to T2 (11 weeks of training), followed by an increase from T2 to T3 (18 weeks of training) and a subsequent decrease at T4 (29 weeks of training).

**Figure 3:**
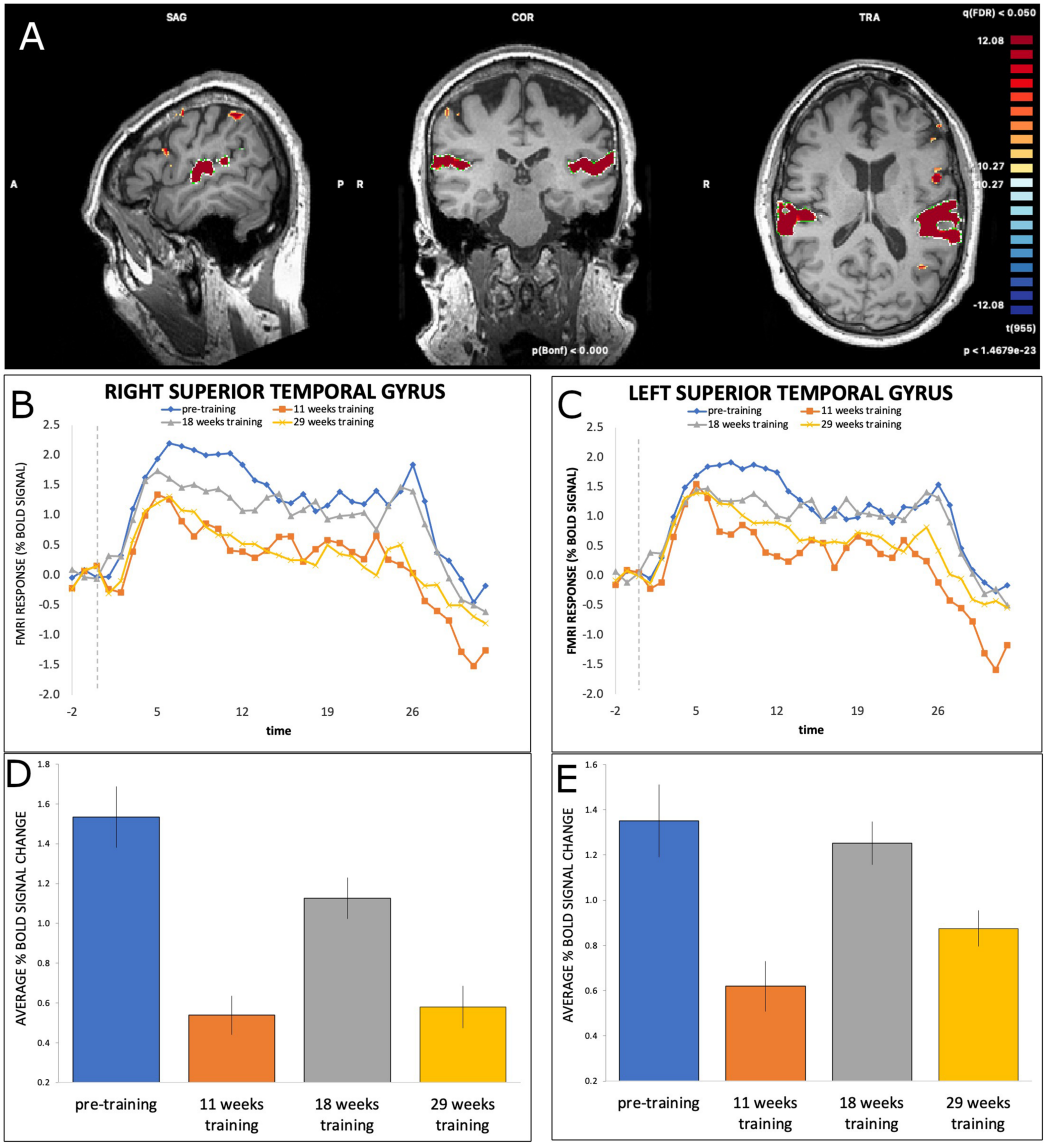
**(A)** BOLD activation of right and left STG during dance-imagery shown in sagittal, coronal, and transverse view in the 3D Talairach space, displayed at a statistical threshold of *p* < 0.0001, Bonferroni-corrected, cluster threshold k > 22; **(B,C)** average percent BOLD signal change of right and left STG during dance-imagery blocks within each of the four timepoints (dashed line indicates the start of the music played in the scanner); **(D,E)** average percent BOLD signal change of right and left STG between the four timepoints. Error bars represent S.E.M.

Significant modulation of BOLD signal was also found for the right STG at T2 (*b* = −1.0, SE = 0.075, *t*(412) = −13.21, *p* < 0.001), T3 (*b* = −0.41, SE = 0.074, *t*(412) = −5.49, *p* < 0.001), and T4 (*b* = −0.95, SE = 0.074, *t*(412) = −12.84, *p* < 0.001), and the left STG at T2 (*b* = −0.73, SE = 0.074, *t*(413) = −9.87, *p* < 0.001) and T4 (*b* = −0.48, SE = 0.073, *t*(413) = −6.51, *p* < 0.001), but not T3 (*b* = −0.10, SE = 0.073, *t*(413) = −1.35, *p* = 0.18). As shown in Figure 3, the changes in activation between timepoints for both right and left STG followed a similar pattern to the SMA, with an initial decrease from T1 to T2 followed by an increase from T2 to T3 and then a decrease at T4.

**Figure 4:**
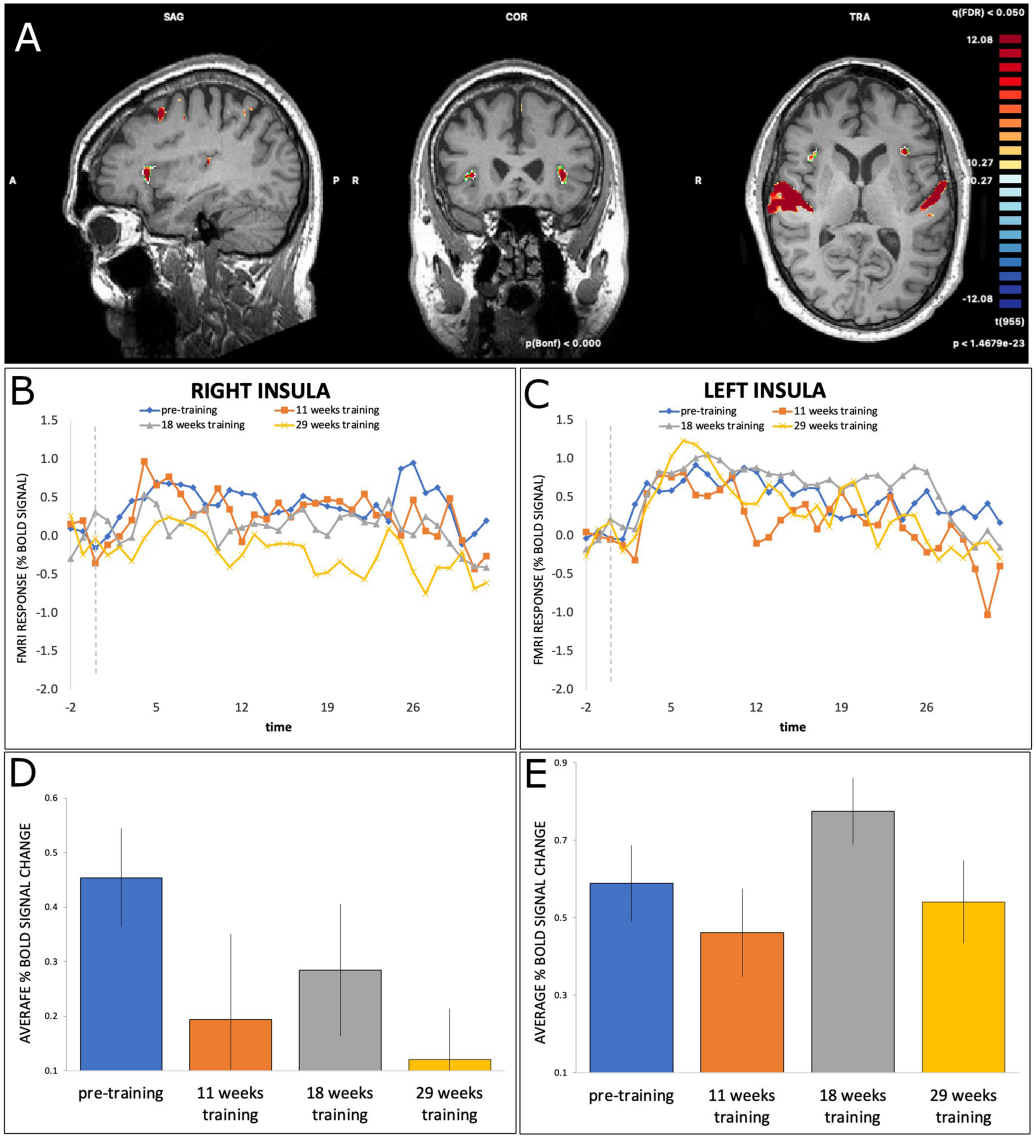
**(A)** BOLD activation of right and left insula during dance-imagery shown in in sagittal, coronal, and transverse view in the 3D Talairach space, displayed at a statistical threshold of *p* < 0.0001, Bonferroni-corrected, with cluster threshold k > 22; **(B,C)** average percent BOLD signal change of right and left insula during dance-imagery blocks within each of the four timepoints (dashed line indicates the start of the music played in the scanner); **(D,E)** average percent BOLD signal change of right and left insula between the four timepoints. Error bars represent S.E.M.

The right insula also showed a similar pattern (Figure 4), with a significant effect of Time at T2 (*b* = −0.26, SE = 0.075, *t*(415) = −3.46, *p* = 0.0006) and T4 (*b* = −0.33, SE = 0.074, *t*(415) = −4.49, *p* < 0.001), but not at T3 (*b* = −0.17, SE = 0.074, *t*(415) = −2.28, *p* = 0.023). Finally, the left insula did not show any significant effects of Time (all *p* > 0.004).

## 4 Discussion

The findings of the present case study suggest that, in an individual with mild PD, long-term dance training promoted functional changes in cortical regions while imagining the dance learned during classes.

The SMA, which is implicated in processes of motor planning, preparation and imagery (Shima and Tanji, 1998; Shima and Tanji, 2000; Lotze and Halsband, 2006), was significantly activated during all four timepoints. The SMA has been found to be activated in people with PD during motor imagery (Cunnington et al., 2001; Weiss et al., 2015), to a similar degree as in healthy controls (Cunnington et al., 2001). In previous work applying the same paradigm as the present study to expert dancers, activations were found in SMA and primary motor cortex (M1) (Bar and DeSouza, 2016), further indicating the role of motor processes during imagined dance. The high initial level of activation in the SMA at T1, when the participant had minimal experience of the music and choreography, might reflect the novelty of the music and/or difficulty in generating motor imagery. Consistent with the latter point, neural activity in people with PD when performing an implicit test of motor imagery (hand laterality judgment) has previously been found to increase with task difficulty in areas including the SMA (Helmich et al., 2007). At T2, a decrease in SMA activity was observed, following 11 weeks of dance classes learning the choreography alongside additional imagery practice with the music at home. This decrease might indicate a reduction in the demands of generating imagery, since the dance choreography was now familiar but not fully learned. The initial pattern of decreasing activation in SMA from T1 to T2 is also consistent with the findings of Olshansky et al. (2015), where decreased activation in this region was found during motor imagery with familiar music compared to unfamiliar music. The pattern of activation also suggested a subsequent increase in SMA activation at 18 weeks into training (T3), which might reflect greater generation of imagery as learning progressed (e.g., more sustained or more detailed images), followed by a decrease at the final timepoint (T4). In expert dancers, a decrease in SMA activation was found after 34 weeks of learning a new choreography (Bar and DeSouza, 2016), indicating greater ease or efficiency of motor imagery over time. In previous investigations of music and familiarity, the SMA and auditory regions were found to be activated when music was familiar, together with other motor-related regions such as the basal ganglia, cerebellum, and dorsal premotor cortex, further suggesting a pattern of motor-related activations (Zatorre et al., 2007). Additionally, recent self-report data indicated that music can evoke motor imagery in people with PD (Poliakoff et al., 2023), and that the vividness of music-evoked motor imagery correlated with musical training and the urge to dance.

The present analysis did not examine functional activity in the pre-SMA, which is a subregion of the SMA located rostral to the SMA-proper. While both areas are involved in motor planning and sequencing (Shima and Tanji, 1998; Shima and Tanji, 2000), the pre-SMA may be more involved in learning new motor sequences, due to its connections with prefrontal areas that are involved in learning and performing movement sequences (Shima and Tanji, 1998; Shima and Tanji, 2000; Hikosaka et al., 1996). The pre-SMA may also be more involved in inhibition of action (Obeso et al., 2013), compared to the SMA-proper which has more dorsal connections. The function of the pre-SMA may also be more impacted in PD than the SMA-proper (Cunnington et al., 2001). Future studies could thus attempt to differentiate the effects of dance training on activity of the SMA-proper and the pre-SMA in people with PD.

The bilateral STG, which have a key role in auditory processing (Rivier and Clarke, 1997) showed a similar pattern of activity to the SMA across the four timepoints, which was also similar to findings from expert dancers (Bar and DeSouza, 2016). In the present study, 29 weeks of training with the same music and choreography promoted an overall decrease in the STG activation in both hemispheres. One possible explanation of this is that novel music strongly activates auditory regions, and this activation decreases with familiarity (Olshansky et al., 2015).

The right and left insula were activated across all timepoints in the present study, but only the right insula showed a significant change across time. In humans, the insula has connections with neural structures such as the frontal, parietal, and temporal lobes, the cingulate gyrus, amygdala, brainstem, thalamus, and basal ganglia (Flynn, 1999). Multiple functions of the insula have been proposed, including sensorimotor processes such as body awareness, error awareness, attention to pain, and representing the physiological condition of the body (Craig, 2009). In addition, the insula has been proposed to provide an interface between body awareness and movement (Tinaz et al., 2018).

The present study found an asymmetry in activation between right and left insula, which may be due to the differences between ascending and descending connections that utilize different frequency bands depending on feedforward or feedback communication (Bastos et al., 2015) or anatomical connections (Rivier and Clarke, 1997). The right insula has been found to be more activated by visual and auditory perception of emotional music (Petrini et al., 2011) as well as processing of rhythm (Lappe et al., 2013) and melody (Thaut et al., 2014) compared to the left. Moreover, the right insula has been suggested to be important for multisensory integration (Chen et al., 2015) and in the present study may have been involved in integrating sensory aspects of the imagined movement with the music. The insula has also been suggested to act as a hub for connecting attentional control and memory related regions (Mayer et al., 2007); thus, it is also possible that the decrease in activation of the right insula in the present study reflects reduced demands on attentional processing, emotion processing and/or memory. Further evidence for a role of the insula in dance was found in a study of older adults (Rehfeld et al., 2018), in which dance training was associated with an increase in grey matter in various brain regions including the left insula, when compared to an active control group who practiced repetitive movements. These findings contrast somewhat with the present study, where a clearer change in activation was found for the right than left insula. However, the previous study emphasized continual learning of different movements and choreographies, which may differently recruit the insula; for example, the left insula appears to be more involved in speech and language processing (Oh et al., 2014), which may be important for following instructions for new dance routines. Moreover, we did not analyze structural changes, which may reveal different effects than functional data.

While previous studies of dance for PD have not examined neuroplastic effects in the same regions as investigated in the present study, some evidence of functional neural changes resulting from dance training has been reported in PD as noted above (Kashyap et al., 2021; Batson et al., 2014). Neuroplastic effects of other forms of physical activity, such as aerobic exercise and treadmill training, have also been documented in people with PD (Duchesne et al., 2016; Maidan et al., 2017; Johansson et al., 2020), although one study found that activation of sensorimotor areas and cerebellum did not change during imagined gait following a 12-week program of either Tango or treadmill training (Myers et al., 2018). In addition, evidence of functional changes in brain areas related to motor imagery, alongside improved motor imagery vividness, was found following training with action observation and motor imagery in participants with PD (Sarasso et al., 2023). Since dance involves observation and imagery of movement (Bek et al., 2020), the neural changes indicated by the present study may thus reflect both motor learning of the dance choreography and improved motor imagery ability.

The present study has several limitations that should be addressed in future work to further understand the neural effects of dance for people with PD. Although the results of a single case study cannot be generalized, this study provides proof of concept of a dance learning paradigm that can be applied to larger numbers of participants to investigate neuroplastic effects of dance. Future studies should compare participants receiving dance training to participants in a control condition, such as an exercise program or no intervention. It would also be informative to collect longitudinal data on performance or recall of the dance choreography, as well as disease status (e.g., UPDRS) to examine in relation to changes in functional activation. In the present study, it cannot be ruled out that stress or anxiety contributed to the high levels of BOLD activity observed at the first timepoint, and this could be addressed in future studies by incorporating physiological measures such as skin conductance and heart rate. Additionally, although the present study provides initial evidence that regular dance participation could promote neuroplasticity in people with PD, it is important to note that the participant in this case study might not be representative of a typical person with PD, given his high level of physical activity including additional dance practice (both physical and via imagery) outside of classes. Moreover, given the heterogeneous nature of PD, the neural effects of dance may differ between participants at different disease stages or with different symptom profiles. Future research with larger samples could investigate this by comparing groups of participants with different subtypes of PD. Alternatively, heterogeneity could be better controlled by focusing only on one subtype, as some previous fMRI studies have done (Sarasso et al., 2023). Finally, since difficulties with motor imagery are sometimes reported in PD (Scarpina et al., 2019; Readman et al., 2023), future studies could also screen participants to ensure an adequate level of imagery ability, as well as providing training on the use of motor imagery prior to undertaking the fMRI protocol.

## 5 Conclusion

In conclusion, the findings of this case study indicate the potential neural effects of dance for people with PD, through activation of multiple brain networks associated with movement, planning, imagery, and auditory and emotional processing.

## Data Availability

The raw data supporting the conclusions of this article will be made available by the authors, without undue reservation.
