# Supplementary material for: Neural effects of multisensory dance training in Parkinson’s disease: evidence from a longitudinal neuroimaging single case study
Source: Front Aging Neurosci. 2024 Oct 9;16:1398871. doi: 10.3389/fnagi.2024.1398871 (PMC11496053; doi:10.3389/fnagi.2024.1398871)
Supplement: Supplementary file 1 [file Data_Sheet_1.pdf]

Table S1. Dance for Parkinson's class sample routine. The exact structure and content of the classes varied over the 8-month training period, but the choreography tested in the fMRI protocol was always practiced at the end of each class.

Part	Description
Opening	Participants introduced themselves by stating names accompanied by a dance movement.
Warm-up	Stretching arms and legs as well as knees and ankles to facilitate flexibility and mobility while also strengthening the core.
Exercise	While seated, participants slowly rise to practice safely rising from a seated position. This is followed by a paired mirror dance, where the participants improvise movements and copy each other. Participants then practice their balance by lunging side to side while holding onto the back of a chair.
Dancing	Participants dance to the triplet rhythm of waltz. Participants also build confidence by performing a seated shuffle dance of gradually increasing expression of dance movements. Finally, participants perform the "*Showdown Hoedown*" choreography facing a partner, comprising a 2-minute choreography with multiple steps and movement sequences.

Table S2. Talairach coordinates of functionally defined regions of interest. *Note: SMA* = supplementary motor area; *STG* = superior temporal gyrus.

Region	Hemisphere	Talairach Coordinates			Number of voxels	Max stat-value
		x	***y***	***z***		
SMA		-3	6	55	4535	16.071
STG	Right	56	-19	11	8084	26.882
	Left	-50	-24	13	7895	25.750
Insula	Right	22	14	1	605	14.245
	Left	-32	20	5	403	13.043
